# Intramolecular hydrogen bonds in 1,4-dihydropyridine derivatives

**DOI:** 10.1098/rsos.180088

**Published:** 2018-06-13

**Authors:** M. Petrova, R. Muhamadejev, B. Vigante, G. Duburs, Edvards Liepinsh

**Affiliations:** Latvian Institute of Organic Synthesis, Aizkraukles 21 Street, Riga 1006, Latvia

**Keywords:** NMR, DFT, 1, 4-dihydropyridines, hydrogen bond, H/D ^13^C isotope effects, IR

## Abstract

1,4-Dihydropyridine (1,4-DHP) derivatives have been synthesized and characterized by ^1^H, ^13^C, ^15^N nuclear magnetic resonance (NMR) spectroscopy, secondary proton/deuterium ^13^C isotope shifts, variable temperature ^1^H NMR experiments and quantum-chemical calculation. The intramolecular hydrogen bonds NH⋯O=C and CH⋯O=C in these compounds were established by NMR and quantum-chemical studies The downfield shift of the NH proton**,** accompanied by the upfield shift of the ^15^N nuclear magnetic resonance signals, the shift to the higher wavenumbers of the NH stretching vibration in the infrared spectra and the increase of the ^1^J(^15^N,^1^H) values may indicate the shortening of the N–H bond length upon intramolecular NH⋯O=C hydrogen bond formation.

## 1. Introduction

**Scheme 1.**
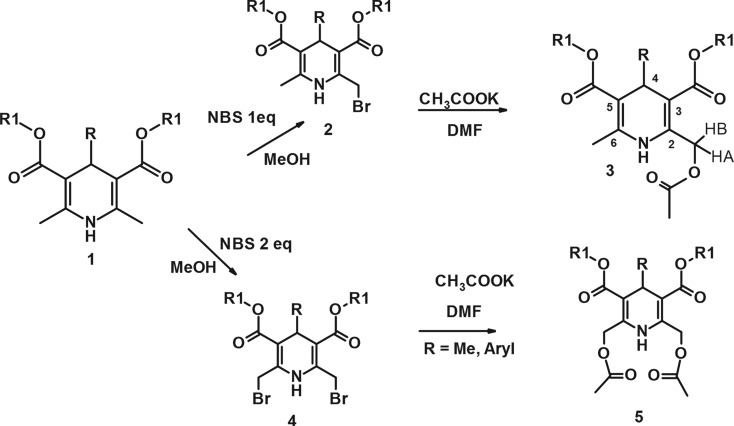
Synthesis of compounds **2–5**. **1a–5a** R = Me; R_1_ = Et; **1a′–3a′** R = Me; R_1_ = Me; **1b–5b** R = Ph; R_1_ = Et; **1c–5c** R = o-CHF_2_-Ph, R_1_ = CH_2_CH_2_O-Pr-n; **3d** R = o-F-Ph, R1 = Et; **3e** R = m-NO_2_-Ph, R_1_ = Et.

Since the original discovery of amlodipine, the 2-substituted 1,4-dihydropyridines have attracted considerable attention owing to their various biological activities [1]. For the aimed synthesis of novel therapeutic agents, it is important to establish what structural factors influence their biological activity. To this end, the modification of 2,6-Me groups was performed. The original method was elaborated for obtaining both alkyl 4-substituted 2-acetoxymethyl-(**3**) and 2,6-bis-acetoxymethyl-1,4-dihydropyridine-3,5-dicarboxylates (**5**) (scheme 1).

Non-covalent interactions have fundamental roles in supramolecular chemistry, drug design, protein folding, crystal engineering and other areas of molecular science. Hydrogen bond interactions are principal forces, which determine the molecular recognition and self-assembly processes as well as the structure of a great variety of chemical and biological systems [2]. The structures of the novel compounds **3** and **5** are interesting because there could be different types of intramolecular interactions.

The investigated compounds **1**, **3** and **5** can be divided into three groups according to their structure. In the first group (**1**) any intramolecular hydrogen NH⋯O bonds are absent, the second one (**3**)—could have one NH⋯O bond and the third group (**5**) may have two such bonds. The NH chemical shift (CS) in ^1^H nuclear magnetic resonance (NMR) spectra in general reflects the strength of the intramolecular hydrogen bond [3]. Additionally, intramolecular hydrogen bonding can be characterized by means of secondary deuterium isotope effects NH(D) on ^13^C NMR chemical shifts [4] as well as Fourier transform infrared (FTIR) spectroscopy [5]. Quantum-chemical calculations should give insight for energetic and geometrical parameters of the intramolecular hydrogen bond in the optimal conformations of the molecules.

## 2. Material and methods

### 2.1. General

All reagents were purchased from Acros, Aldrich, Alfa Aesar or Merck and used without further purification. Thin layer chromatography was performed on silica gel 60 F254 aluminium sheets 20 × 20 cm (Merck), as eluent using (20% EtOAc/hexane). The purities of compounds were determined by high performance liquid chromatography on a Waters Alliance 2695 system and Waters 2489 ultraviolet–vis detector equipped with Alltima C18 column (5 µm, 4.6 × 150 mm, Grace) using a gradient elution with acetonitrile/phosphoric acid (0.1%) in water, at a flow rate of 1 ml min^−1^. Peak areas were determined electronically with a Waters Empower 2 chromatography data system.

### 2.2. Nuclear magnetic resonance spectroscopic experiments

The one-dimensional ^1^H- and ^13^C- and two-dimensional ^1^H-^1^H nuclear overhauser effect spectroscopy (NOESY), ^13^C-^1^H heteronuclear multiple bond correlation (HMBC), ^13^C-^1^H heteronuclear single-quantum correlation (HSQC) spectra of compounds **1–5** were recorded on a Varian-Mercury 400 MHz, Varian-400mr 400 MHz, Varian UNITY INOVA 600 MHz and Bruker Avance III HD 800 MHz, in CDCl_3_, at temperature 25°C.

^15^N NMR spectra were recorded at 60.81 MHz (Varian UNITY INOVA 600 MHz) spectrometer equipped with a cryoprobe and at 81.10 MHz (Bruker Avance III HD 800 MHz). Spectra were acquired using HSQC standard pulse sequences provided by Varian or Bruker Biospin spectrometer libraries. The magnetization from ^1^H to ^15^N in HSQC spectra were transferred using coupling constants ^1^*J*(^15^N,^1^H) = 95. ^15^N spectra were indirectly referenced to liquid ammonia.

### 2.3. Infrared spectroscopic experiments

The infrared (IR) spectra were obtained in the fine films on the Shimadzu IR Prestige 21 FTIR spectrometer.

### 2.4. Elemental analysis experiments

Instrumental elemental analysis is based on method of ‘flash combustion’ Elemental analyzer Carlo Erba mod. EA-1108.

### 2.5. Compounds

Dialkyl 4-aryl-2-acetoxymethyl-6-methyl-1,4-dihydropyridine-3,5-dicarboxylates are scarcely investigated; they were mainly obtained by Hantzsch cyclization using commercially not available ethyl 4-acetoxy-3-oxobutanoate [6] or in a four-step procedure from appropriate 2-dietoxymethyl-1,4-DHP [7].

We propose the two steps procedure from dimethyl 4-substituted 2,6-dimethyl-1,4-dihydropyridine-3,5-dicarboxylate *via* bromination of 2-methyl- or 2,6-dimethyl groups.

**Figure 1.**
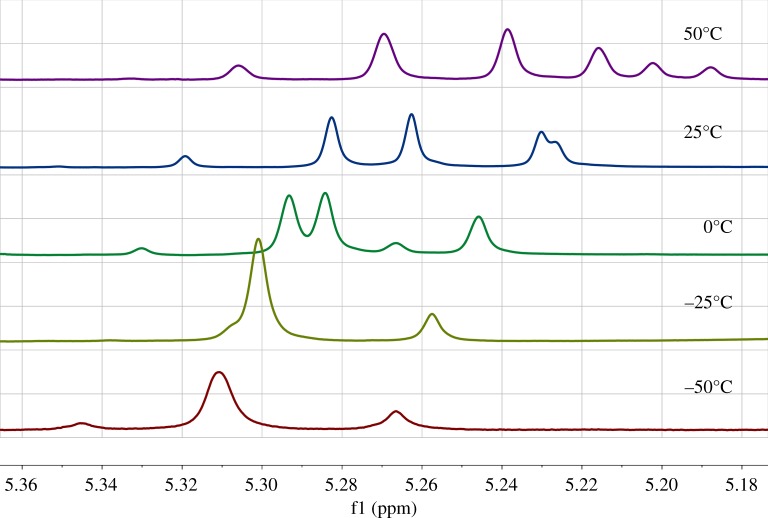
Temperature dependence of the AB system ^1^H chemical shifts for **3a** in CDCl_3_.

The necessary 2-bromomethyl-1,4-DHP **2** and 2,6-bis-bromomethyl-1,4-DHP **4** were obtained by our elaborated method including the bromination of the methyl groups in the position 2- (or 2,6-) with N-bromosuccinimide (NBS) in methanol according to the literature [8,9]. The further reaction of appropriate monobromomethyl-1,4-dihydropyridine (DHP) **2** or bis-bromomethyl-1,4-DHP **4** with dry potassium acetate in dry dimethylformamide (DMF) lead to target compounds **3** and **5** in medium-to-good yields (figure 1).

### 2.6. General procedure for the synthesis of 4-substituted diethyl(dimethyl) 2-acetoxymethyl-6-methyl-1,4-dihydropyridine-3,5-dicarboxylates **3**

Appropriate 4-substituted ethyl(methyl) 2-bromomethyl-6-methyl-1,4-dihydropyridine-3,5-dicarboxylate **2** (0.9 mmol, 1 eq) was dissolved in dry DMF (3 ml) under argon. Anhydrous potassium acetate (97 mg, 0.99 mmol, 1.1 eq) was added at 0°C. The reaction mixture was stirred at room temperature for 20 h. The mixture was diluted with water (10 ml) and extracted with EtOAc, the organic layer washed with water and brine and dried over Na_2_SO_4_. After filtration and evaporation, the crude residue was purified by column chromatography (silica gel, eluent—chloroform/petroleum ether (bp 40–60°C)/acetone (9 : 7 : 1), giving 2-acetoxymethyl-1,4-DHP **3** as powder or oil.

#### 2.6.1. Diethyl 2-acetoxymethyl-4,6-dimethyl-1,4-dihydropyridine-3,5-dicarboxylate (**3a**)

Yield: 0.22 g 74%; yellowish oil. Mass spectrometry (MS) (+electrospray ionization (ESI)) *m/z* (relative intensity) 326 ([M + H]^+^ 60).

#### 2.6.2. Dimethyl 2-acetoxymethyl-4,6-dimethyl-1,4-dihydropyridine-3,5-dicarboxylate (**3a′**)

Yield: 0.15 g 56%; white powder; m.p. 78–83°C. MS (+ESI) *m/z* (relative intensity) 298 ([M + H]^+^ 45). Anal. Calc. for C_14_H_19_NO_6_: C, 56.56; H, 6.44; N, 4.71; found: C, 56.48; H, 6.31; N, 4.69.

#### 2.6.3. Diethyl 2-acetoxymethyl-6-methyl-4-phenyl-1,4-dihydropyridine-3,5-dicarboxylate (**3b**)

Yield: 0.23 g 67%; white powder; m.p. 104–106°C ([10] 106°C. MS (+ESI) *m/z* (relative intensity) 388 ([M + H]^+^ 50). Anal. Calc. for C_21_H_25_NO_6_: C, 65.10; H, 6.50; N, 3.62; found: C, 64.99; H, 6.53; N, 3.60.

#### 2.6.4. Bis-(2-propoxyethyl) 2-acetoxymethyl-4-(2-difluoromethoxyphenyl)-6-methyl-1,4-dihydropyridine-3,5-dicarboxylate (**3c**)

Yield: 0.37 g 72%; yellow oil. MS (+ESI) *m/z* (relative intensity) 570 ([M + H]^+^ 72).

#### 2.6.5. Diethyl 2-acetoxymethyl-4-(2-fluorophenyl)-6-methyl-1,4-dihydropyridine-3,5-dicarboxylate (**3d**)

Yield: 0.22 g 61%; white powder; m.p. 102–104°C. MS (+ESI) *m/z* (relative intensity) 406 ([M + H]^+^ 82). Anal. Calc. for C_21_H_24_ FNO_6_: C, 62.21; H, 5.97; N, 3.45; found: C, 62.02; H, 6.05; N, 3.37.

#### 2.6.6. Diethyl 2-acetoxymethyl-6-methyl-4-(3-nitrophenyl)-1,4-dihydropyridine-3,5-dicarboxylate (**3e**)

Yield: 0.20 g 52%; light yellow powder; m.p. 105–107°C ([11] 106°C. MS (+ESI) *m/z* (relative intensity) 433 ([M + H]^+^ 100). Anal. Calc. for C_21_H_24_ N_2_O_8_: C, 58.33; H, 5.59; N, 6.48; found: C, 58.12; H, 5.65; N, 6.21.

### 2.7. General procedure for the synthesis of 4-substituted diethyl 2,6-bis-acetoxymethyl-1,4-dihydropyridine-dicarboxylates **5**

Appropriate 4-substituted diethyl 2,6-bis-bromomethyl-1,4-dihydropyridine-3,5-dicarboxylate **4** (1.2 mmol, 1 eq) was dissolved in dry DMF (4 ml) under argon. Anhydrous potassium acetate (283 mg, 2.88 mmol, 2.4 eq) was added at 0°C. The reaction mixture was stirred at room temperature for 16 h. The mixture was diluted with water (20 ml) and extracted with EtOAc, the organic layer washed with water and brine and dried over Na_2_SO_4_. After filtration and evaporation, the crude residue was crystallized from dilute methanol giving 2,6-bis-acetoxymethyl-1,4-DHP **5**.

#### 2.7.1. Diethyl 2,6-bis-acetoxymethyl-4-methyl-1,4-dihydropyridine-3,5-dicarboxylate (**5a**)

Yield: 0.37 g 81%; light yellow crystals; m.p.91°C. MS (+ESI) *m/z* (relative intensity) 385 ([M + H]^+^ 100). Anal. Calc. for C_18_H_25_NO_8_: C, 56.39; H, 6.57; N, 3.65; found: C, 56.08; H, 6.60; N, 3.49.

#### 2.7.2. Diethyl 2,6-bis-acetoxymethyl-4-phenyl-1,4-dihydropyridine-3,5-dicarboxylate (**5b**)

Yield: 0.40 g 75%; light yellow crystals; m.p.128–130°C. MS (+ESI) *m/z* (relative intensity) 446 ([M + H]^+^ 82). Anal. Calc. for C_23_H_27_NO_8_: C, 62.01; H, 6.11; N, 3.14; found: C, 61.85; H, 6.25; N, 3.10.

#### 2.7.3. Bis-(2-propoxyethyl) 2,6-bis-acetoxymethyl-4-(2-difluoromethoxyphenyl)-1,4-dihydropyridine-3,5-dicarboxylate (**5c**)

Yield: 0.44 g 58%; white crystals; m.p.76–78°C. MS (+ESI) *m/z* (relative intensity) 628 ([M + H]^+^ 100). Anal. Calc. for C_30_H_39_ F_2_ NO_11_: C, 57.41; H, 6.26; N, 2.23; found: C, 57.11; H, 6.35; N, 2.08.

### 2.8. Computational details

All calculations were performed in Gaussian 09 [12] using the hybrid density functional theory (DFT) B3LYP functional with basis set 6–311++G(d,p) with empirical dispersion D3 version of Grimme with Becke-Johnson damping (GD3BJ) [13]. All geometries were optimized using the polarizable continuum model (PCM) solvation model for chloroform. For all optimized conformers, vibrational harmonic frequencies were calculated at the same level of theory, all the obtained harmonic frequencies were positive. For chemical shift calculation a gauge-independent atomic orbital (GIAO) method was used.

## 3. Results and discussion

The structures of **1**, **3**, **5** were analysed by multinuclear ^1^H, ^13^C, ^15^N NMR spectroscopy (tables 1 and 2) and quantum-chemical methods.

**Table 1.** ^1^H,^15^N NMR data for 1,3,5 in CDCl_3_.

	*δ*(^1^HN), ppm	*δ*(H_A_), ppm	^1^*J*(^13^C,^1^H_A_), Hz	*δ*(H_B_), ppm	^1^*J*(^13^C,^1^H_B_), Hz	^2^*J*(H,H), Hz	*Δδ*(^1^HN)/T, ppb K^−1^	*δ*(^15^N), ppm	^1^*J*(^15^N,^1^H), Hz
**1a**	5.54						−5.6	134.53	92.4
**1b**	5.67						−6.1	134.01	93.6
**1c**	5.73						−10.4	134.36	93.6
**3a**	6.48	5.21	153	5.27	155	14.7	−3.2	122.90	93.3
**3a′**	6.53	5.25	153	5.29	155	14.7	−3.0	125.42	93.0
**3b**	6.55	5.27	153	5.40	156	14.8	−2.9	124.12	93.6
**3c**	6.64	5.26	154	5.32	155	14.8	−2.8	122.69	93.6
**3d**	6.57	5.27	154	5.39	156	14.9	−2.8	123.31	94.8
**3e**	6.67	5.32	154	5.37	156	14.9	−2.5	124.25	94.2
**5a**	7.55	5.23	153	5.36	156	15.3	−3.6	113.14	94.8
**5b**	7.65	5.24	154	5.44	157	15.2	−3.6	114.18	94.8
**5c**	7.77	5.25	154	5.35	156	15.2	−3.6	115.38	95.6

**Table 2.** ^13^C Chemical shifts, (ppm) in **1**, **3**, **5**.

	COO···H	C_6_	C_2_	C_5_	C_3_	C_6_-Me	C_2_-CH_2_	C_4_	C_4_Me
**1a**		144.28	144.28	104.63	104.63	19.44		28.49	22.22
**1b**		144.39	144.39	103.79	103.79	19.28		39.62	
**1c**		144.96	144.96	102.35	102.35	19.22		35.88	
**3a**	170.82	144.10	141.76	105.81	104.49	19.50	61.37	28.54	22.05
**3a′**	170.85	144.43	142.07	105.46	104.25	19.48	61.28	28.43	22.00
**3b**	170.76	143.47	141.35	105.05	104.15	19.67	61.41	39.60	
**3c**	170.71	144.16	142.03	103.28	102.52	19.70	61.33	36.22	
**3d**	170.71	143.88	141.82	103.76	103.01	19.60	61.31	34.18	
**3e**	170.72	144.36	142.27	103.99	103.32	19.76	61.24	39.83	
**5a**	170.75	141.95	141.95	105.21	105.21		61.25	28.58	22.08
**5b**	170.70	141.44	141.44	104.52	104.52		61.26	39.54	
**5c**	170.59	142.23	142.23	102.83	102.83		61.23	36.19	

### 3.1. ^1^H, ^15^N nuclear magnetic resonance and infrared spectra

The X-ray structures of 3,5-mono- and 3,5-dicarboxylates of 1,4-DHP as well as quantum-chemical calculations [6] show that the carbonyl groups adopt the *s-cis, s-cis* conformation relatively to C_3_=C_2_ or C_6_=C_5_ double bonds. This is unusual, because it is well known that for any dienes *s-trans* orientation is preferable, owing to the better conjugation. The reason for the peculiar *s-cis* conformation of the fragment –C=C–C=O is not clear. However, in the case of 1,4-DHP derivatives it was proposed that the *s-cis* orientation is favoured by weak intramolecular CH⋯O=C interaction [7]. Such interactions are possible in **1**, **3**, **5** too.

The signals of the methylene groups of the alkoxy carbonyl chains in position 3 and 5 for compounds **3** and **5** show a rather complex splitting pattern, owing to the presence of a prochiral centre on C_4_ leading to the diasteretopicity of the two hydrogens of the methylene group. The rest of the signals in ^1^H NMR spectra are in agreement with the nature of aromatic or aliphatic hydrogen atoms (table 1).

The two methylene hydrogens of the substituents on C_2_ and C_6_ in **3** and **5** are also diastereotopic and they give an AB system at 5.24–5.44 ppm in the ^1^H NMR spectra. The non-equivalence of these protons is characterized by the ^1^H chemical shift difference (*δ*(H_A_) − δ(H_B_)) which may be affected by such factors as the conformation of the 1,4-DHP ring, the anisotropic influence of the substituents, the hindered rotation of the substituents at C_2,6_ and the intramolecular hydrogen bond of the type CH⋯O. The geminal constants ^2^*J*(H_A_,H_B_) slightly increase from 14.8 Hz in **3** to 15.2 Hz in **5** (table 1). For the compounds **3** the difference in the chemical shifts of the AB methylene protons varies in the range of 0.043–0.130 ppm depending on the R substituents at C_4_.

^1^H NOESY spectra of compounds **3** and **5**, allow us to determine the relative position of the AB protons for the methylene groups—the NOE from NH to the more shielded protons H_A_ is more intense than the NOE to H_B_. This means that the H_A_ proton is located closer to the NH proton and cannot form a CH⋯O = C hydrogen bond.

The ^1^H NMR signals of these protons show unusual temperature dependence (figure 1) similarly to 4-aryl-2,6-bis-(bromo-methyl)-1,4-DHP [14]. The temperature dependence of the AB quartet excludes an ordinary exchange process, because the signals do not exhibit exchange line broadening prior to coalescence and the chemical shifts of the both protons vary simultaneously. This is in contradiction with classical dynamic NMR theories [15].

**Figure 2.**
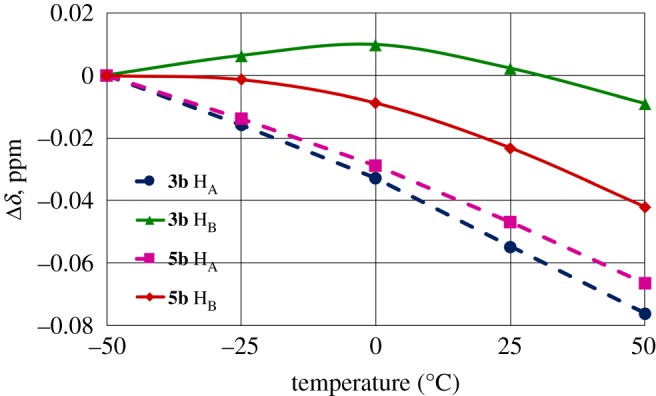
Temperature dependence of the C_2,6_-CH_2_ methylene protons chemical shifts for **3b** and **5b** in CDCl_3_. Solid and dashed lines represent the values for H_B_ and H_A_ protons, respectively. The chemical shift at 0°C is taken as zero point for each proton. **3b** H_A_ (filled circles), **3b** H_B_ (filled triangles), **5b** H_A_ (filled squares) and **5b** H_B_ (filled diamonds).

At temperature variation, the CS of the H_A_ proton in **3** and **5** changes linearly with the same coefficient as the CS of 2-CH_3_ methyl protons in **1** (table 1). So, the H_A_ proton should be available to the solvent (chloroform), to the same extent as in **1**. At the same time the signal of H_B_ changes nonlinearly and the curve is close to a negative parabolic (figure 2). The nonlinear dependence (*δ*(H_B_) versus T) can point to the fact that H_B_ is engaged in a weak intramolecular hydrogen bond C–H⋯O=C.

The unusual temperature influence on the diastereotopic methylene proton signals could be explained by changes in the conformer ratio owing to two conformational processes: the rotation of the substituent around the C_2,6_─CH_2_ and around the C_3,5_─CO_2_ bonds. These processes have relatively high barriers for transitions between the possible conformations (approximately 16–18 kcal mol^−1^ (66.94–75.31 kJ mol^−1^) and approximately 8–10 kcal mol^−1^ (33.47–41.84 kJ mol^−1^), respectively) and unusual temperature effects on the methylene proton signals are caused by changes in the conformer ratio. So, the observed AB proton resonances are the statistically averaged resonance signals of the various conformations, the populations of which are temperature dependent [14].

The measured coupling constants ^1^*J*(^13^C,^1^H) for each of the methylene AB protons are different (table 1). The larger ^1^*J*(^13^C,^1^H) value corresponds to the less shielded proton H_B_. This is consistent with the literature data, because if the CH proton is involved in a hydrogen bond the ^1^*J*(^13^C,^1^H) value usually increases [16]. Indeed, in our previous work [9] the hydrogen bonded protons were characterized by the larger value of the ^1^*J*(^13^C,^1^H). On the basis of the data obtained it can be concluded that the intramolecular CH⋯O=C hydrogen bond could be one of the reasons for the non-equivalence of the methylene protons in these systems and conformations with s-*cis*-s-*cis* carboxyl group orientation relative to the double bond in the dihydropyridine cycle.

The ^1^H NMR spectra show a broad signal of the NH proton in the intervals, 5.54–5.70 ppm (**1**), 6.55–6.68 ppm (**3**) and 7.65–7.77 ppm (**5**) (table 1).

**Figure 3.**
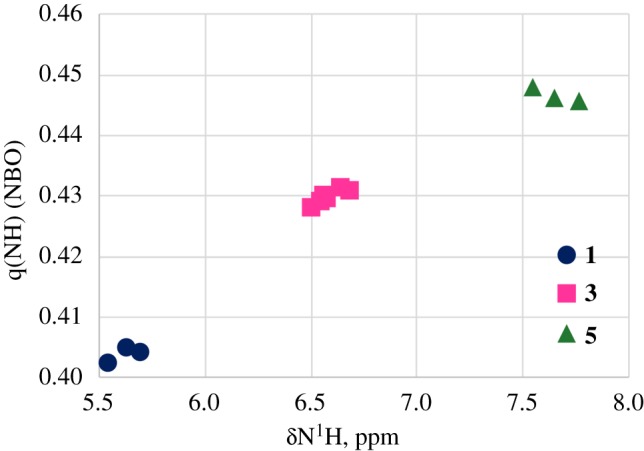
A plot of the NBO charges (q) versus *δ*(^1^HN) in **1**(filled circles)–**3**(filled squares)–**5**(filled triangles).

The low field shift of the N^1^H proton signal in the sequence **1**, **3**, **5**, may reflect the formation of an intramolecular N–H⋯O hydrogen bond in compounds **3** and **5** between the N^1^H proton and the oxygen atoms of the OCOMe substituents at C_2_ and C_6_ carbons [17,18]. On the other side the low-field shift of N^1^H resonance could correspond partly to the decrease of the electron density on N^1^H atom as calculated by the B3LYP-NBO method in the sequence **1**, **3**, **5** (figure 3).

The temperature coefficient (Δ*δ*/ΔT) of the N^1^H signal is usually used to detect the presence of intramolecular H-bonds [18,19]. The breaking of hydrogen bonds, induced by increasing the temperature, moves the resonance signal of N^1^H upfield. The temperature coefficients with the values more positive than −4.5 ppb K^−1^ are strongly indicative of intramolecular H-bonds.

The temperature coefficients of the NH proton resonance in **1**, **3**, **5** have been measured recording the ^1^H NMR spectra in CDCl_3_ in the temperature range 223–323 K (table 1). The obtained values fall between −2.5 and −10.4 ppb K^−1^. The NH protons of **3** have the Δ*δ*/ΔT values more positive than −4.5 ppb K^−1^ (approx. −3 ppb K^−1^) which indicates the involvement of these NH protons in the intramolecular H-bonding. In compounds **5,** that have two acetoxymethyl substituents and, consequently, may have two hydrogen bonds, the Δ*δ*/ΔT values are lower than in **3** (approx. −3.6 ppb K^−1^). This could be the consequence of the weaker NH⋯O=C hydrogen bond in case of **5** when compared to **3**.

**Figure 4.**
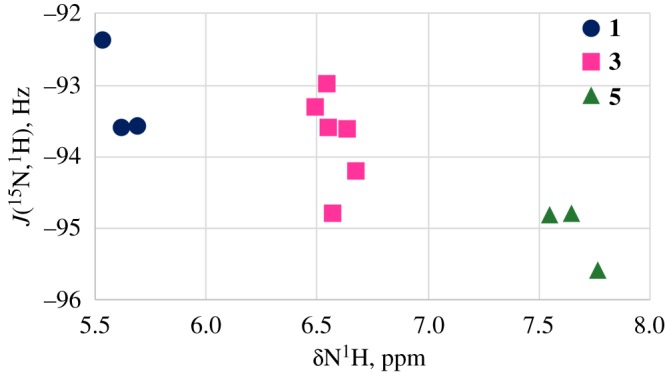
A plot of ^1^*J*(^15^N,^1^H) versus N^1^H chemical shifts in **1**(filled circles)–**3**(filled squares)–**5**(filled triangles).

The absolute values of the measured coupling constants ^1^*J*(^15^N,^1^H) 92–96 Hz slightly increase in the sequence **1**, **3**, **5** (figure 4) and suggest that the hydrogen is stably bonded to the nitrogen atom and there is little intermolecular exchange, this is in contradiction, because increased positive charge on the NH proton (deshielding) should decrease the absolute value of the ^1^*J*(^15^N,^1^H) [20,21]. However, when the nitrogen of the proton donor is *sp*^2^ hybridized the changes in ^1^*J(*^15^N,^1^H) are quadratically related to the length of the corresponding NH distances of nitrogen bases: the shorter *d*(NH) corresponds to the larger ^1^*J*(^15^N,^1^H) value [22]. This could point to the slight decrease of the NH distance in the sequence **1**–**3**–**5**.

According to [23] if NH participate in the N–H⋯X hydrogen bond, the ^1^*J*(^15^N,^1^H) coupling is affected by mainly two factors, the electrostatic effect and the n(X) → (N–H)* charge transfer interaction. The first one is known to cause an increase in the absolute value of ^1^*J*(^15^N,^1^H) and the second leads to a decrease in the absolute value of ^1^*J*(^15^N,^1^H) coupling. Their influence on the IR stretching frequency of the N–H bond are also opposite to each other, so the charge transfer interactions correspond to a red shift while the electrostatic effect corresponds to a blue shift. Usually, hydrogen bonds lead to an elongation of the X–H covalent bond and its stretching vibration *ν*_XH_ shifts to the lower wavenumbers (red shift) [5,18]. The shift of *ν*(NH) to the higher wavenumbers on hydrogen bonding probably is accompanied by the shortening of the NH distance (so-called blue shift). Such a kind of effect has been registered for the C–H and N–H bonds previously [24].

**Figure 5.**
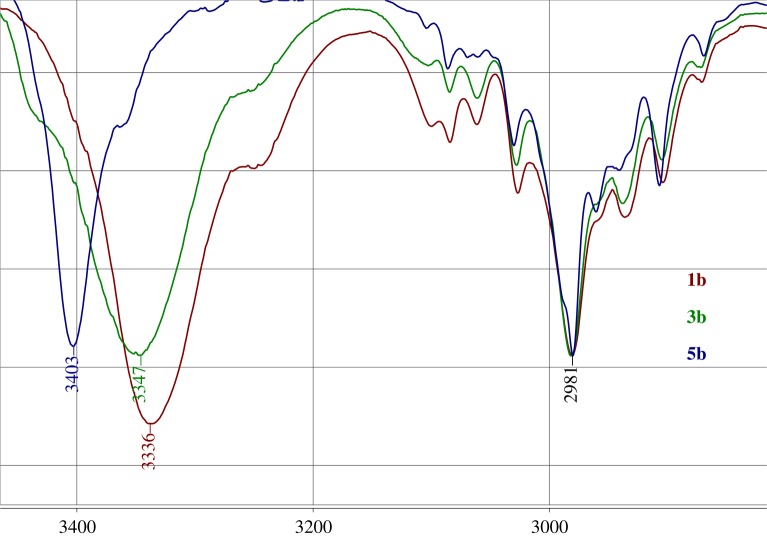
N–H and C–H stretching frequencies for **1b**, **3b** and **5b**, cm^−1^.

The IR spectral data of **1a–c** with no hydrogen bonds are characterized by the stretching *ν*(NH) bands in the range 3344–3336 cm^−1^. The NH groups engaged in one hydrogen bond in compounds **3a–c** display the NH-stretching vibration in the interval 3347–3348 cm^−1^ and the NH groups engaged in two hydrogen bonds in compounds **5a–c** display the *ν*(NH) bands in the range 3363–3437 cm^−1^ (figure 5). The *ν*(NH) band moves up to 8 cm^−1^ on going from **1b** to **3b**, and up to 64 cm^−1^ on going from **1b** to **5b**. The IR stretching *ν*(NH) band frequencies and the ^1^*J(*^15^N,^1^H) values in compounds **1–5** indicate that both of these parameters (*ν*(NH), ^1^*J*(^15^N,^1^H)) could be partly caused by the same reason—the difference in the length of the NH bond [25].

The ^15^N HMQC spectra with the value of the direct NH coupling constants (^1^*J*(^15^N,^1^H) = 95 Hz) were used to measure the chemical shift for the nitrogen nucleus [26,27]. The ^15^N resonance signals appear in three distinct regions: 134.53–134.01 ppm in **1**, 123.31–124.42 ppm in **3** and 114.18–115.58 ppm in **5 **ppm (table 1).

**Figure 6.**
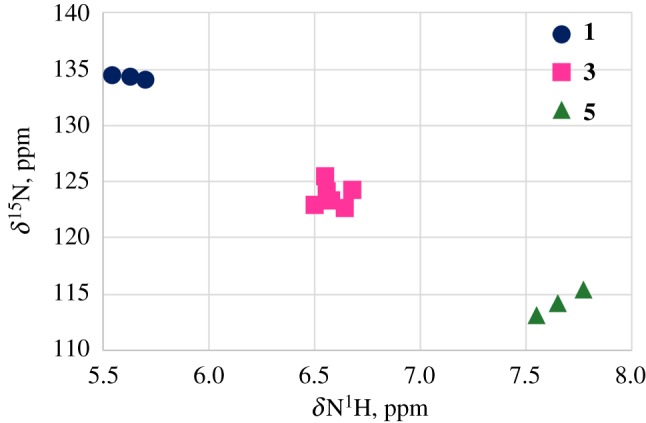
The relationship between the ^15^N chemical shifts and ^1^H chemical shift in **1(**filled circles**)–3(**filled squares**)–5(**filled triangles**)**.

**Figure 7.**
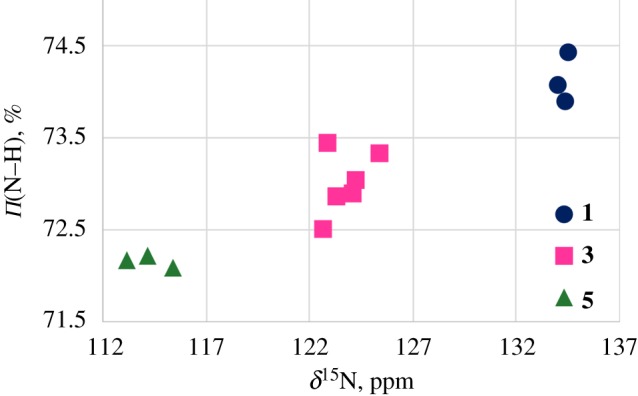
The relationship between ^15^N chemical shifts and the *π*-components of the N–H bonds calculated by NBO method for **1**(filled circles)–**3**(filled squares)–**5**(filled triangles).

The downfield shift of the NH proton signal is accompanied by the upfield shift of the ^15^N signal in the sequence **1**, **3**, **5** (figure 6). This is surprising because usually the shielding of the N^1^H proton is accompanied by the shielding of the adjacent nitrogen atom [28]. Commonly the NH bond elongates on hydrogen bond formation [29]. According to [30] the observed ^15^N CS of amide nitrogens move upfield with a shortening of the N–H bond in the C=O···H–N hydrogen bond. So, the reverse dependence *δ*(^15^N)/*δ*(^1^H) in the sequence **1**, **3**, **5** (figure 7) may probably indicate the shortening of the N–H bond, but cannot quantitatively explain a significant upfield shift of the ^15^N resonance signal on going from **1**–**3**–**5**

In the data obtained by the natural bond orbital (NBO) method, the average π-orbital character of the N atom in NH-bond decreases in the sequence **1**(74.2%)–**3**(73.0%)–**5**(72.2%) (figure 7). That should lead to a diamagnetic shift of the ^15^N-resonance signal.

**Figure 8.**
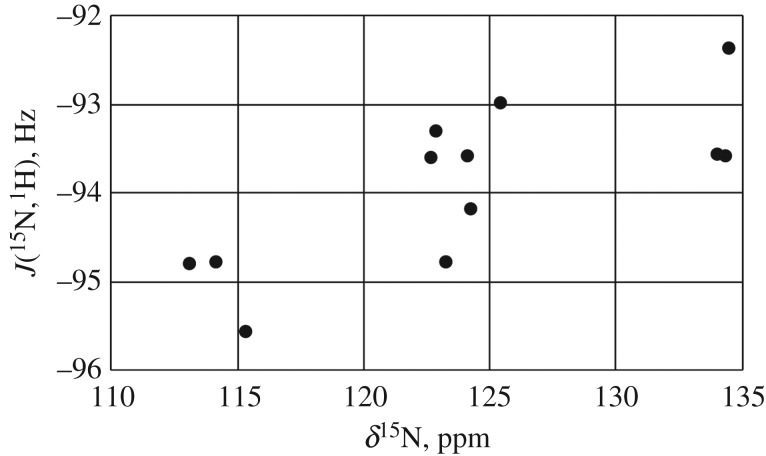
The correlation between ^1^*J*(^15^N,^1^H) values and *δ*^15^NH chemical shifts in compounds **1**, **3** and **5**.

A relationship between the ^15^N chemical shifts and the ^1^*J(*^15^N,^1^H) values in compounds **1**, **3** and **5** (figure 8), reflects that both of these parameters (*δ*(^15^N), ^1^*J*(^15^N,^1^H)) could be caused partly by the same reason—the difference in the length of the NH bond [25].

So, the *δ*(^1^H) downfield shift and the upfield shift for *δ*(^15^N) in the sequence **1**–**3**–**5** partly can be accompanied by the decrease of the N–H bond length. This is supported by the increase of the ^1^*J(*^15^N,^1^H) absolute value in compounds **1**, **3** and **5** (figure 8). A very popular explanation of diamagnetic shift of heavy nuclei is sterical perturbation of the electronic cloud nearby. In our case this may be owing to the spatial affinity between the acetoxy substituent and ^15^N nucleus. According to the literature data [31] such effect could shift ^15^N resonance signal up to 10 ppm to higher fields.

### 3.2. ^13^C nuclear magnetic resonance spectra

The ^13^C NMR spectra of compounds **1**, **3**, **5** display signals in the carboxyl, aromatic and aliphatic regions (table 2). The ^13^C resonances in the 1,4-DHP ring (table 2) are relatively insensitive to the nature of the substituents on C_3_, C_4_ and C_5_. The ^13^C NMR spectra of compounds **1**, **3**, **5** show the signals for C_3_ and C_5_ atoms at the lower values (ca 102–104 ppm), than those expected for the typical olefinic carbons atoms, while the C_2_ and C_6_ signals appear at higher values (approx. 141–145 ppm). These findings have been accounted for by the strong push–pull effect of the groups linked to the olefinic double bonds like ones previously observed in other related systems. The carbon signal of C_4_ is in agreement with the previous data [32,33] and appears at 28.5 ppm for the aliphatic R and in the interval 34.2–39.8 ppm when R is aromatics.

### 3.3. Proton/deuterium isotope effects on ^13^C chemical shifts

Measurement of the secondary ^n^Δ^13^C(proton/deuterium (H/D)) deuterium isotope effects (IE) is known to be a very good tool in studying intramolecular hydrogen bonds [4]. In compounds **1**, **3**, **5** the replacement of H by D at the nitrogen atom produces only intrinsic isotope effects, because the values of ^1^*J*(^15^N,^1^H), approximately 92–96 Hz, allow us to neglect any equilibrium processes. Intrinsic isotope effects are of vibrational origin and are owing to the anharmonicity of the potential curve of the N–H⋯X bond. Hydrogen bonding increases this anharmonicity. The changes in *δ*(^13^C) are the result of the shortening of the NH/D bond upon deuteration [34].

The values of IE (*^n^*Δ^13^C(H/D) = *δ*(^13^C(NH)) − *δ*(^13^C(ND))) have been measured from the ^13^C spectra of partly deuterated samples where the signals of the two isotopomers could be easily identified on the basis of their relative intensity. All measured isotope effects in **1**, **3**, **5** were positive (table 3).

**Table 3.** N(H/D) isotope effects on ^13^C atoms for **1**–**3**–**5**, ppb.

	COO···H	C_6_	C_2_	C_5_	C_3_	C_6_-Me	C_2_-CH_2_	C_4_Me
**1a**		85	85	50	50	81	—	26
**1b**		84	84	51	51	82	—	—
**1c**		90	90	47	47	84	—	—
**3a**		86	78	59	44	84	37	30
**3a′**		88	79	59	45	84	37	28
**3b**	3	87	80	59	42	84	36	—
**3c**	5	96	88	58	41	84	36	—
**3d**	5	94	87	58	41	84	35	—
**3e**	4	91	85	57	43	84	37	—
**5a**	15	82	82	56	56	—	32	27
**5b**	18	85	85	55	55	—	33	—
**5c**	18	91	91	53	53	—	34	—

The substituent effects on IE values are mainly described by two factors: the hydrogen bond and the transmission of the electronic effects of substituents [35].

The N(H/D) isotope effects on the acetoxymethyl carbonyl ^13^C atoms have been registered (table 3). For compounds **3** where the NH atom is involved in one hydrogen bond, the ^5^Δ^13^C_COO_ (H/D) values are in the range 3–5 ppb. At the same time the ^5^Δ^13^C_COO_ (H/D) values at the carboxyl carbon for **5** 3–5 times exceed (approx. 15–18 ppb) them for **3**. Probably, this is owing to some difference in NH⋯O hydrogen bonding in **3** and **5**.

If we consider the influence of the N(H/D) replacement on the ^13^C resonances inside the 1,4-DHP cycle (table 3) there is little change in the sequence **1**–**3**–**5**. This corresponds to the small change of the ^13^C chemical shifts (table 2). Interestingly, that the N(H/D) IE on the C_2,6_-^13^CH_3_ atom of the methyl groups separated from the N^1^H proton by three bonds, is similar to the effect on the ^13^C_2,6_ carbons separated by only two bonds. At the same time, the N(H/D) IE values for the ^13^C_3,5_ atoms are much smaller (table 3). The ^13^C isotope shifts of the methyl groups at C_2_ and C_6_ in **1** and **3** are considerably larger than the corresponding values of the methylene groups at the same atoms in compounds **3** and **5** (table 3).

No ^13^C(NH/D) isotope effects are observed on the C_4_ carbon atom in the 1,4-DHP cycle, however in 4-Me substituted compounds **1a**, **3a** and **5a** the positive long-range isotope effects ^5^ΔC_4−Me_ (H/D) approximately 26–28 ppb are observed. The observation of such a long-range isotope effect at the methyl group C_4_-CH_3_ is rather unusual. Since there is no general theory about the long-range IE we have problems in interpretation of the long ranges ^13^C IE observed in **1**, **3**, **5** compounds.

### 3.4. Density functional theory calculations

In compounds **3** and **5** there could be four possible types of the intramolecular H-bonds (NH⋯O=, NH⋯-O–, CH⋯O = and CH⋯–O–) defining the optimal configuration of the molecules.

To establish which of the available types of bonds is dominant in stabilizing the optimal geometry of compounds **3** and **5**, the quantum-chemical calculations of the possible conformations of compounds **3a′** and **5a** were performed.

**Figure 9.**
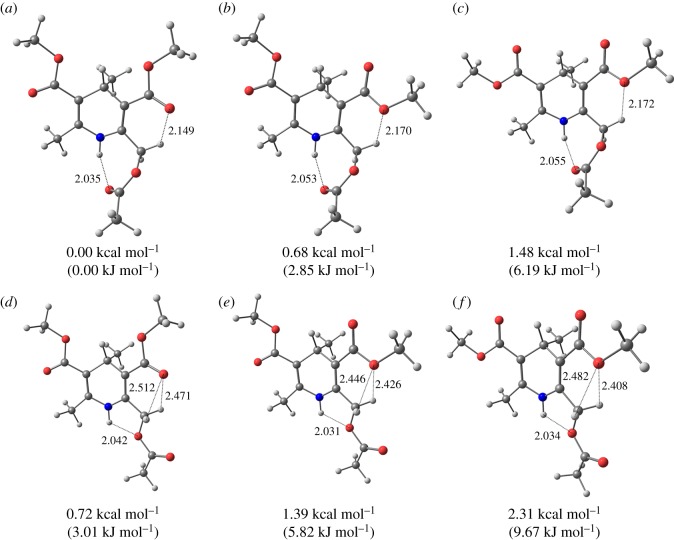
(*a*–*f*) The most stable conformations of **3a’**.

**Figure 10.**
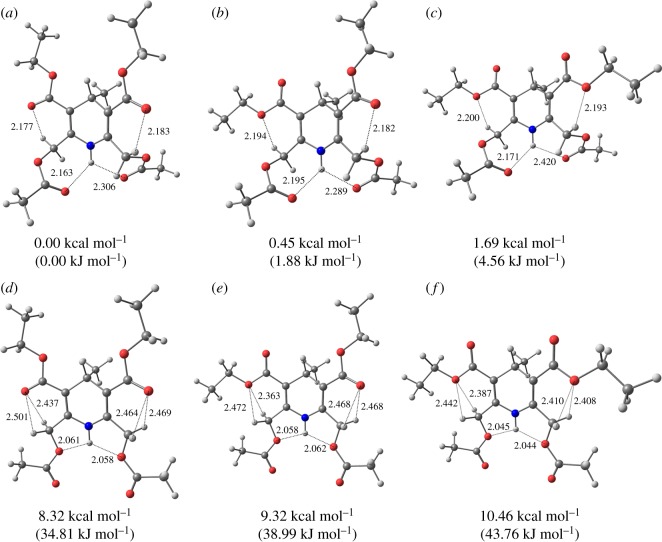
(*a*–*f*) The most stable conformations of **5a**.

The most stable conformations of **3a′** and **5a** and the calculated geometric parameters of the intramolecular hydrogen bonds CH⋯O and NH⋯O are presented in figures 9 and 10 and table 4.

**Table 4.** Intramolecular H-bonds parameters for the preferred conformations of **3a′** and **5a**.

compound (conformation)	C_3_–C = O, C_5_C = O orientation	energy, kcal mol^−1^ (kJ mol^−1^)	*d*, Å (*α*,°) NH⋯O = C	*d*, Å (*α*,°) NH⋯–O–	*d*, Å (*α*,°) CH⋯O = C	*d*, Å (*α*,°) CH⋯–O–
**3a′ (A)**	*s-cis, s-cis*	0.00 (0.00)	2.035 (141.3)		2.149 (123.4)	
**3a′ (B)**	*s-trans, s-cis*	0.68 (2.85)	2.053 (140.1)			2.170 (115.8)
**3a′ (C)**	*s-trans, s-trans*	1.48 (6.19)	2.065 (139.9)			2.172 (115.8)
**3a′ (D)**	*s-cis, s-cis*	0.72 (3.01)		2.042 (109.0)	2.512 (88.8)	
					2.471 (91.0)	
**3a′ (E)**	*s-trans, s-cis*	1.39 (5.82)		2.031 (109.5)		2.446 (88.4)
						2.426 (89.4)
**3a′ (F)**	*s-trans,s-trans*	2.31 (9.67)		2.034 (109.5)		2.482 (86.8)
						2.408 (90.7)
**5a (A)**	*s-cis, s-cis*	0.00 (0.00)	2.163 (130.9)		2.183 (119.2)	
			2.306 (119.7)		2.177 (120.9)	
**5a (B)**	*s-cis, s-trans*	0.45 (1.88)	2.195 (128.9)		2.182 (119.3)	2.194 (112.8)
			2.289 (120.8)			
**5a (C)**	*s-trans, s-trans*	1.09 (4.56)	2.171 (130.5)			2.193 (111.3)
			2.420 (113.7)			2.200 (113.2)
**5a (D)**	*s-cis, s-cis*	8.32 (34.81)		2.058 (107.2)	2.464 (90.8)	
					2.469 (90.6)	
				2.058 (107.2)	2.501 (89.0)	
					2.437 (92.3)	
**5a (E)**	*s-cis, s-trans*	9.32 (38.99)		2.062 (107.3)	2.468 (90.7)	2.472 (86.6)
				2.058 (107.2)	2.468 (90.7)	2.363 (92.4)
**5a (F)**	*s-trans, s-trans*	10.46 (43.76)		2.044 (107.9)		2.410 (89.5)
						2.408 (89.6)
				2.045 (107.8)		2.442 (88.0)
						2.387 (91.0)

In compound **3a′** it is possible to form two intramolecular hydrogen bonds of the type NH⋯O: one with the carbonyl oxygen (conformations **A**, **B**, **C**) and the other with the ether oxygen (conformations **D**, **E**, **F**) of the adjacent 2-acetoxymethyl substituent.

The interaction between the NH hydrogen and the carbonyl oxygen of the carboxylic group (conformations **A**–**C**) when compared to ones with the alkoxy oxygen (structures **D**–**F**) is characterized by the similar geometric and energetic characteristics (table 4.). The most energy preferable conformation of **3a′** is **A**. But the energy difference of **D** and **A** is small and equal to 0.72 kcal mol^−1^ (3.01 kJ mol^−1^). The similar values are observed for the energy difference between **E** and **B** (0.71 kcal mol^−1^ (2.97 kJ mol^−1^)) as well as for **F** and **C** (0.83 kcal mol^−1^ (3.47 kJ mol^−1^)).

The longer distances *d*_NH⋯O_ (2.04–2.07 Å) and the larger angles *α*_NH⋯O_ (139.9–141.3°) in **A–C** are counterbalanced by the shorter values of *d*_NH⋯O_ (2.03–2.04 Å) and the smaller values of *α*_NH⋯O_ (109.0–109.5°) in **D–F**. It is known that the shorter the hydrogen bond length and the larger angle the stronger the hydrogen bond is. Thus, it can be argued that in **3a′** both NH⋯O = and NH⋯–O– hydrogen bonds are about equally probable.

In **5a** the conformations **A**, **B**, **C** in NH⋯O interactions with O=C groups are energetically more preferable than the corresponding **D**, **E**, **F** with NH interacting with alkoxy oxygen. The energy difference is in the interval 8.32–9.37 kcal mol^−1^ (34.81–39.20 kJ mol^−1^).

The geometric characteristic of conformations **A–C** of **5a** are close to each other and are characterized by the longer distances *d*_NH⋯O_ (2.16–2.42 Å) and the larger angles *α*_NH⋯O_ (113.7–130.9°) as compared to **D**–**F** with the shorter values of *d*_NH⋯O_ (2.04–2.06 Å) and the smaller values of *α*_NH⋯O_ (107.1–107.9°).

The stability of the NH⋯O hydrogen bond depends on the 3-(5-) side chain orientation and decrease in the following sequence: *s-cis, s-cis* (**A**, **D**); *s-cis, s-trans* (**B**, **E**), *s-trans, s-trans* (**C**, **F**).

The optimal conformations of the side chain 3,5-carbomethoxy groups were shown before to be *s*-*cis*, *s-cis* [9,26]. The obtained results reveal that in **3a’** the energy minima correspond to the conformations with *s-cis-s-cis* carboxyl group orientation (**A**, **D**) relative to the double bond in the dihydropyridine cycle and there is a possibility to form the C_2,6_H···O bonds with the oxygens of the carboxyl groups at C_3_ and C_5_. These additional H-bonds stabilize the molecule, as the structures with *s-cis, s-trans* (**B**, **E**) and *s-trans, s-trans* (**C**, **F**) carboxyl groups orientation have the higher energy.

According to the calculations, the distances *d*(CH⋯O) increase, and the angles *α*(C-H⋯O) decrease on going from **A**–**C** to **D**–**F**, indicating weakening of the CH⋯O hydrogen bonds between the methylene protons and the oxygens of the carboxyl groups. At the same time in conformations **A**–**C** only one methylene proton participates in the CH⋯O hydrogen bond, and in **D**–**E** conformations both methylene protons form CH⋯O bonds. Although the first one is stronger because it is characterized by the shorter distance *d*_CH⋯O_ and the larger angle *α*_CH⋯O_, two weaker CH⋯O bonds in **D**–**E** might have the same stabilizing effect for the molecule optimal conformations. This tendency is true for any orientations of the carbomethoxy groups at C_3_, C_5_ positions.

In **5a** the optimal conformation **A** is characterized by *s-cis, s-cis* O=C 3,5-carboxyl group orientation relative to the double bond in the dihydropyridine cycle and the NH⋯O hydrogen bond with two O=C oxygens (**A**).

The structures with *s-cis-s-trans* (**B**) and *s-trans-s-trans* (**C**) orientation of the 3,5-carboxyl groups in **5a** similarly to **3a′** have the higher energy.

The calculations of 3,5-side chain conformations of **5a** show that, the distances *d*(CH⋯O) increase, and the angles (C–H⋯O) decrease on going from **–**-**C** to **D**–**F**, indicating a weakening of the CH⋯O hydrogen bonds between the methylene protons and the oxygens of the carboxyl groups. Similarly to **3a’** in conformations **A–C** of **5a** only one methylene proton participates in the CH⋯O = hydrogen bond, and in **D–E** conformations of **5a** both methylene protons form approximately equal CH⋯–O– bonds. Although the first hydrogen bond is stronger because it is characterized by the shorter distance *d*_CH⋯O_ and the larger angle *α*_CH⋯O_, two weaker CH⋯O bonds in **D**–**E** of **5a** might have the same stabilizing effect on the optimal conformations of the molecule. This tendency is true for any orientations of the carbomethoxy groups at C_3_, C_5_ positions.

In conformations **A**–**C** of the compounds **3a′** and **5a**, the methylene protons at C_2_, C_6_ are nonequivalent, because only one participates in the CH⋯O hydrogen bond. In conformations **D–F** of **3a′** and **5a**, both methylene protons are equivalent because they form two equal CH⋯O hydrogen bonds (table 4). Owing to the energy advantage of the conformation **A** over **D** of compounds **5** and energetic equivalence of **A** and **D** conformations in **3**, the difference of the CS of the methylene protons (*δ*(H_A_)-δ(H_B_)) in **5** is twice as high than in **3**.

The calculations performed demonstrate that in case of compounds **3**, the NH proton could preferably form a 5-membered H-chelate cycle with the alkoxy oxygen. In compounds **5** two 7-membered H-chelate cycles with the C=O oxygens in 2 and 6 substituents are formed. Different types of the NH⋯O bonds in **3** and **5** can be the reason for the increase of the values of the secondary ^n^Δ^13^C(NH/D) isotope shifts in ^13^C NMR spectra on the carboxyl group carbon in substituents at C_2,6_ on going from **3** to **5**. The greater isotope effect of the NH/D substitution through 6 bonds to the ^13^C CS of the carboxylic carbons in substituents at positions 2 and 6 in compounds **5** (^6^Δ^13^C(NH/D)), than through 4 bonds (^4^Δ^13^C(NH/D)) in **3** proves that the NH···O hydrogen bond in the seven-membered H-chelate cycle formed by the C=O group is stronger than the one in the five-membered H-chelate formed by the alkoxy group.

## 4. Conclusion

The ^1^H,^13^C,^15^N NMR, FTIR spectral data and quantum chemical calculations show that alkyl 4-substituted 2-acetoxymethyl- (**3**) and 2,6-bis-acetoxymethyl-1,4-dihydro-pyridine-3,5-dicarboxylates (**5**) form four types of the intramolecular H-bonds (NH⋯O and CH⋯O) defining the optimal structure of the molecule.

^13^C isotope effects induced by N(H/D) substitution registered on the acetoxymethyl carbonyl carbon in compounds **5** are considerably larger than in **3**. That demonstrates that the NH proton interacts differently with oxygens in **3** and **5**.

In monosubstituted compounds **3**, the NH proton preferably forms a five-membered H-chelate cycle with the alkoxy oxygen, while in disubstituted compounds **5**, two 7-membered H-chelate cycles with the carbonyl oxygens of the carboxyl groups in 2- and 6-substituents are preferred.

The downfield shift of the ^1^H NMR signal of the NH proton**,** accompanied by the upfield shift of the ^15^N NMR signals and the increase of the ^1^*J*(^15^N,^1^H) values in the sequence from dimethyl 4-substituted 2,6-dimethyl-1,4-dihydropyridine-3,5-dicarboxylate (**1**) to **3** and **5**, may indicate the shortening of the N–H bond length upon hydrogen bond formation. This is supported by the shift of NH stretching band to the higher wavenumbers in the FTIR spectra of **3** and **5**

The optimal conformers of **3** and **5** with the lowest energy have the 3,5-carbonyl groups in *s-cis, s-cis* position relative to the double bonds of the DHP cycle (C_3_=C_2_ and C_6_=C_5_). They are stabilized by weak intramolecular hydrogen bonds of the CH⋯O type. The diastereotopic protons of the methylene groups at positions C_2_ and C_6_ show unusual temperature dependence due to the changes in the populations of the optimal conformations.

## Data accessibility

Supporting data are available at Dryad (http://dx.doi.org/10.5061/dryad.j3m73) [36].

## References

[1] Hyvönen Z, Plotniece A, Reine I, Chekavichus B, Duburs G, Urtti A 2000 Novel cationic amphiphilic 1,4-dihydropyridine derivatives for DNA delivery. Biochim. Biophys. Acta 1509, 451–466. (doi:10.1016/S0005-2736(00)00327-8) 11118554 10.1016/s0005-2736(00)00327-8

[2] Kuhn B, Mohr P, Stahl M 2010 Intramolecular hydrogen bonding in medicinal chemistry. J. Med. Chem. 53, 2601–2611. (doi:10.1021/jm100087s) 20175530 10.1021/jm100087s

[3] Del Bene JE, Perera SA, Bartlett RJ 1999 Hydrogen bond types, binding energies, and ^1^H NMR chemical shifts. J. Phys. Chem. A 103, 8121–8124. (doi:10.1021/jp9920444)

[4] Dziembowska T, Hansen PE, Rozwadowski Z 2004 Studies based on deuterium isotope effect on ^13^C chemical shifts. Prog. Nucl. Magn. Reson. Spectrosc. 45, 1–29. (doi:10.1016/j.pnmrs.2004.04.001)

[5] Joseph J, Jemmis ED 2007 Red-, blue-, or no-shift in hydrogen bonds: a unified explanation. J. Am. Chem. Soc. 129, 4620–4632. (doi:10.1021/ja067545z) 17375920 10.1021/ja067545z

[6] Meyer H, Scherling D, Karl W 1983 Nitrendipine: identification and synthesis of main metabolites. Arzneimittelforschung. 33, 1528–1534. 6686447

[7] Chang C-C et al. 2010 Antagonism of 4-substituted 1,4-dihydropyridine-3,5-dicarboxylates toward voltage-dependent L-type Ca^2+^ channels Ca_V_1.3 and Ca_V_1.2. Bioorg. Med. Chem. 18, 3147–3158. (doi:10.1016/j.bmc.2010.03.038) 20382537 10.1016/j.bmc.2010.03.038

[8] Petrova M, Muhamadejev R, Cekavicus B, Vigante B, Plotniece A, Sobolev A, Duburs G, Liepinsh E 2014 Experimental and theoretical studies of bromination of diethyl 2,4,6-trimethyl-1,4-dihydropyridine-3,5-dicarboxylate. Heteroat. Chem. 25, 114–126. (doi:10.1002/hc.21145)

[9] Petrova M, Muhamadejev R, Vigante B, Cekavicus B, Plotniece A, Duburs G, Liepinsh E 2011 Intramolecular C-H⋯O hydrogen bonding in 1,4-dihydropyridine derivatives. Molecules 16, 8041–8052. (doi:10.3390/molecules16098041) 21931285 10.3390/molecules16098041 PMC6264772

[10] Bossert FDCD, Wehinger EDCD, Meyer HDCD, Heise AD, Kazda SD, Stoepel KD, Towart RD, Vater WD, Schlossmann KDCD 1978 In 2-position substituierte 1,4- dihydropyridin-derivate, verfahren zu ihrer herstellung sowie ihre verwendung als arzneimittel In the 2-position substituted 1,4-dihydropyridine derivatives, process for their production and their use as drug. Patent no. DE2658183A1.

[11] Satoh Y, Ichihashi M, Okumura K 1991 Studies on Nilvadipine. I. Synthesis and structure-activity relationships of 1,4-Dihydropyridines containing novel substituents at the 2-Position. Chem. Pharm. Bull. (Tokyo) 39, 3189–3201. (doi:10.1248/cpb.39.3189) 1814611 10.1248/cpb.39.3189

[12] Frisch MJ et al. 2013 Gaussian 09, revision D.01. Gaussian 09, revis. Wallingford, CT: D.01, Gaussian, Inc.

[13] Grimme S, Ehrlich S, Goerigk L 2011 Effect of the damping function in dispersion corrected density functional theory. J. Comput. Chem. 32, 1456–1465. (doi:10.1002/jcc.21759) 21370243 10.1002/jcc.21759

[14] Petrova M, Muhamadejev R, Chesnokov A, Vigante B, Cekavicus B, Plotniece A, Duburs G, Liepinsh E 2014 Spectral and quantum-chemical study of nonequivalence of methylene protons in 1,4-Dihydropyridine derivatives*. Chem. Heterocycl. Compd 49, 1631–1639. (doi:10.1007/s10593-014-1414-6)

[15] Günther H 2013 NMR spectroscopy: basic principles, concepts and applications in chemistry. New York, NY: John Wiley & Sons.

[16] Marshall JL 1983 Carbon-Carbon and carbon-proton Nmr couplings: applications to organic stereochemistry and conformational analysis. Deerfield Beach, FL: Verlag Chemie International.

[17] Becker ED 2007 Hydrogen bonding. In *Encyclopedia of Magnetic Resonance*, Chichester, UK: John Wiley & Sons, Ltd.

[18] Gellman SH, Dado GP, Liang GB, Adams BR 1991 Conformation-directing effects of a single intramolecular amide-amide hydrogen bond: variable-temperature NMR and IR studies on a homologous diamide series. J. Am. Chem. Soc. 113, 1164–1173. (doi:10.1021/ja00004a016)

[19] Baxter NJ, Williamson MP 1997 Temperature dependence of ^1^H chemical shifts in proteins. J. Biomol. NMR 9, 359–369. (doi:10.1023/A:1018334207887) 9255942 10.1023/a:1018334207887

[20] Dingley AJ, Masse JE, Peterson RD, Barfield M, Feigon J, Grzesiek S 1999 Internucleotide scalar couplings across hydrogen bonds in Watson–Crick and Hoogsteen base pairs of a DNA triplex. J. Am. Chem. Soc. 121, 6019–6027. (doi:10.1021/ja9908321)

[21] Ishikawa R, Kojima C, Ono A, Kainosho M 2001 Developing model systems for the NMR study of substituent effects on the N-H⋯N hydrogen bond in duplex DNA. Magn. Reson. Chem. 39, S159–S165. (doi:10.1002/mrc.941) 10.1093/nass/1.1.9 12836238

[22] Del Bene JE, Elguero J 2006 Systematic ab Initio Study of 15 N– 15 N and 15 N– ^1^H spin–spin coupling constants across N–H^+^ –N hydrogen bonds: predicting N–N and N–H coupling constants and relating them to hydrogen bond type. J. Phys. Chem. A 110, 7496–7502. (doi:10.1021/jp0613642) 16759141 10.1021/jp0613642

[23] Contreras R 2000 Angular dependence of spin–spin coupling constants. Prog. Nucl. Magn. Reson. Spectrosc. 37, 321–425. (doi:10.1016/S0079-6565(00)00027-3)

[24] Hobza P, Havlas Z 2000 Blue-shifting hydrogen bonds. Chem. Rev. 100, 4253–4264. (doi:10.1021/cr990050q) 11749346 10.1021/cr990050q

[25] Contreras RH, Peralta JE, Giribet CG, Ruiz de azúa MC, Facelli JC 2000 Advances in theoretical and physical aspects of spin-spin coupling constants. Ann. Rep. NMR Spectrosc 41, 55–184. (doi:10.1016/S0066-4103(00)41009-4)

[26] Goba I, Turovska B, Belyakov S, Liepinsh E 2014 Synthesis, spectroscopic and conformational analysis of 1,4-dihydroisonicotinic acid derivatives. J. Mol. Struct. 1074, 549–558. (doi:10.1016/j.molstruc.2014.06.044)

[27] Goba I, Liepinsh E 2013 15 N NMR of 1,4-dihydropyridine derivatives. Magn. Reson. Chem. 51, 391–396. (doi:10.1002/mrc.3959) 23696534 10.1002/mrc.3959

[28] Kyogoku Y 1981 Application of 15 N NMR spectroscopy to studies of the intermolecular interaction of biomolecules. Appl. Spectrosc. Rev. 17, 279–335. (doi:10.1080/05704928108060407)

[29] Bagheri S, Masoodi HR, Abadi MN 2015 Estimation of individual NH⋯X (X = N, O) hydrogen bonding energies in some complexes involving multiple hydrogen bonds using NBO calculations. Theor. Chem. Acc. 134, 127 (doi:10.1007/s00214-015-1738-z)

[30] Kuroki S, Ando S, Ando I, Shoji A, Ozaki T, Webb GA 1990 Hydrogen-bonding effect on 15N NMR chemical shifts of the glycine residue of oligopeptides in the solid state as studied by high-resolution solid-state NMR spectroscopy. J. Mol. Struct. 240, 19–29. (doi:10.1016/0022-2860(90)80492-3)

[31] Witanowski M, Stefaniak L, Webb GA. 1982 Nitrogen NMR Spectroscopy. In Annual reports on NMR spectroscopy (ed. Webb GA), pp. 1–486. Cambridge, MA: Academic Press.

[32] Suárez M, Molero D, Salfrán E, Rodríguez H, Coro J, Sáez E, Martínez-Álvarez R, Martín N 2011 NMR study of 1,4-dihydropyridine derivatives endowed with long alkyl and functionalized chains. J. Braz. Chem. Soc. 22, 166–171. (doi:10.1590/S0103-50532011000100022)

[33] Liepin'sh EE, Zolotoyabko RM, Chekavichus BS, Sausin’ AE, Lusis VK, Dubur GY 1989 13C-NMR spectra of substituted 1,4-dihydropyridines. Chem. Heterocycl. Compd. 25, 1032–1037. (doi:10.1007/BF00487304)

[34] Sobczyk L, Obrzud M, Filarowski A 2013 H/D isotope effects in hydrogen bonded systems. Molecules 18, 4467–4476. (doi:10.3390/molecules18044467) 23591926 10.3390/molecules18044467 PMC6269986

[35] Buncel E, Jones JR. 1991 *Isotopes in the physical and biomedical sciences: isotopic applications in NMR studies*. Amsterdam, The Netherlands: Elsevier Science Publishers.

[36] Petrova M, Muhamadejev R, Vigante B, Duburs G, Liepinsh E 2018 Data from: Intramolecular hydrogen bonds in 1,4-dihydropyridine derivatives Dryad Digital Repository. (http://dx.doi.org/10.5061/dryad.j3m73) 10.1098/rsos.180088 PMC6030305 30110409

