# Correction to ‘Intramolecular hydrogen bonds in 1,4-dihydropyridine derivatives’

**DOI:** 10.1098/rsos.180990

**Published:** 2018-07-18

**Authors:** M. Petrova, R. Muhamadejev, B. Vigante, G. Duburs, Edvards Liepinsh

*R. Soc. Open Sci*. **6**, 180088 (Published 13 June 2018) (doi:10.1098/rsos.180088)

This correction refers to the figure citation in section 2.5. Compounds:

Figure 10 has been incorrectly cited in place of scheme 1.

## 2.5. Compounds

Dialkyl 4-aryl-2-acetoxymethyl-6-methyl-1,4-dihydropyridine-3,5-dicarboxylates are scarcely investigated; they were mainly obtained by Hantzsch cyclization using commercially not available ethyl 4-acetoxy-3-oxobutanoate [6] or in a four-step procedure from appropriate 2-diethoxymethyl-1,4-DHP [7].

We propose the two steps procedure from dimethyl 4-substituted 2,6-dimethyl-1,4-dihydropyridine-3,5-dicarboxylate *via* bromination of 2-methyl- or 2,6-dimethyl groups.

The necessary 2-bromomethyl-1,4-DHP **2** and 2,6-bis-bromomethyl-1,4-DHP **4** were obtained by our elaborated method including the bromination of the methyl groups in the position 2- (or 2,6-) with *N*-bromosuccinimide (NBS) in methanol according to the literature [8,9]. The further reaction of appropriate monobromomethyl-1,4-dihydropyridine (DHP) **2** or bis-bromomethyl-1,4-DHP **4** with dry potassium acetate in dry dimethylformamide (DMF) lead to target compounds **3** and **5** in medium-to-good yields (scheme 1).

